# Prevalence, sociodemographic distribution, treatment and control of diabetes mellitus in Panama

**DOI:** 10.1186/1758-5996-5-69

**Published:** 2013-11-13

**Authors:** Anselmo J Mc Donald P, Jose A Montenegro G, Clara E Cruz G, Aida L Moreno de Rivera, Alberto Cumbrera O

**Affiliations:** 1 Gorgas Memorial Institute for Health Research, Justo Arosemena Avenue and 35th Street, Panama, Republic of Panama; 2 Ministry of Health, Santo Tomas Hospital. Endocrinology Service, Balboa Avenue and 34th East Street, Panama, Republic of Panama; 3 School of Statistics, Faculty of Sciences. University of Panama, Transisthmian Avenue, Panama, Republic of Panama

**Keywords:** Prevalence of diabetes mellitus in Panama, Sociodemographic distribution of diabetes mellitus, Treatment and control of diabetes mellitus

## Abstract

**Background:**

To estimate the prevalence, socio-demographic distribution, treatment and control of diabetes mellitus in Panama.

**Methods:**

A cross-sectional, descriptive study was conducted in the provinces of Panama and Colon, applying a survey on cardiovascular risk factors and analyzing biochemical indicators in 3590 persons. A single-stage, probabilistic, and randomized sampling strategy with a multivariate stratification was used. Individuals with a previous medical diagnosis of diabetes, glycemia ≥ 126 mg/dl and/or glycosylated hemoglobin ≥ 6.5% (≥ 48 mmol/mol) were considered with diabetes mellitus. The prevalence estimates were calculated as percentages with 95% confidence intervals and a p value. Logistic regression was used to identify the sociodemographic variables that were significantly associated with diabetes. Odds ratio and p values were calculated using 2 x 2 tables, and a value of p ≤ 0.05 was considered statistically significant.

**Results:**

Of the participants, 7.3% (262/3590) were aware of having diabetes and 2.2% (78/3590) were unaware. The estimated prevalence of diabetes mellitus was 9.5% (340/3590) and increased in proportion to increasing age. The logistic regression revealed relationships between diabetes and age, sex, area of residence and sociocultural groups. 77.9% of the people aware of having diabetes received treatment and 53.4% have not stabilized the disease.

**Conclusions:**

The research evidenced a high prevalence of diabetes mellitus in Panama, where being Afro-Panamanian and 50 years of age or older are sociodemographic risk factors for DM. Due to the complications that the disease may present we recommend actively searching for such cases to increase diagnosis of people unaware of having diabetes.

## Background

Diabetes Mellitus (DM), particularly type 2, which accounts for 90% - 95% of all cases [1-3], has become a global health problem [4]. It is estimated that by the year 2030, there will be 552 million people affected by this disease, an increase of 50.8% from the 366 million cases recorded in 2011 [5].

It is considered equivalent to coronary heart disease in its public health impact [6] because it shortens life expectancy and deteriorates quality of life. Worldwide, it is also one of the leading causes of blindness [2,3,7,8], chronic kidney failure [2,3,8-10], amputations [2,3,8,11] and other complications [2,3], all of which lead to significant disability and increase health care costs.

A patient with DM, given the multiple complications of the disease, incurs significant health care costs. In Spain, for example, the cost per patient is 1,305 EUR per year [12], and in the United States, the hospitalization costs associated with diabetes total $12 billion dollars per year [13].

Because DM is usually a silent disease in its initial stages [14], only a proactive search for it will lead to a timely diagnosis. In the United States, for example, it is estimated that 26.7% of people with diabetes are unaware that they suffer from the disease [15].

For this reason, type 2 DM is often diagnosed relatively late (between 4–7 years after the disease initially developed) [14]. Therefore, when patients are diagnosed, many have already developed chronic complications from the disease. In the United Kingdom’s Prospective Diabetes Study, of the patients with type 2 DM, 25% had retinopathy, 9% had neuropathy and 8% had nephropathy at the time of the diagnosis [16].

In most countries, DM is among the top ten causes of death [17]. In Panama, according to the National Institute of Statistics and Census (INEC for its acronym in Spanish), DM was the fifth leading cause of death in 2011, representing 5.5% (836/15240) of the total number of registered deaths [18].

Increases in population growth, aging of a population and urban development are social determinants associated with an increase in the prevalence of DM [19]. Thus, population health surveys are important because they can determine the potential impact of sociodemographic variables on the epidemiologic behavior of this disease.

Until 2012, there were no epidemiological indicators about DM in the Panamanian population. This article provides new information about this region and aims to estimate the prevalence, sociodemographic distribution, treatment and control of diabetes mellitus in Panama.

The analysis in this study used the findings from the first Survey on Risk Factors Associated with Cardiovascular Disease (PREFREC for its acronym in Spanish), which was conducted with individuals 18 years and older between October 2010 and January 2011 by the Gorgas Memorial Institute for Health Research (GMI) and the Panamanian Ministry of Health (MOH).

## Methods

### Research design and area

**Figure 1:**
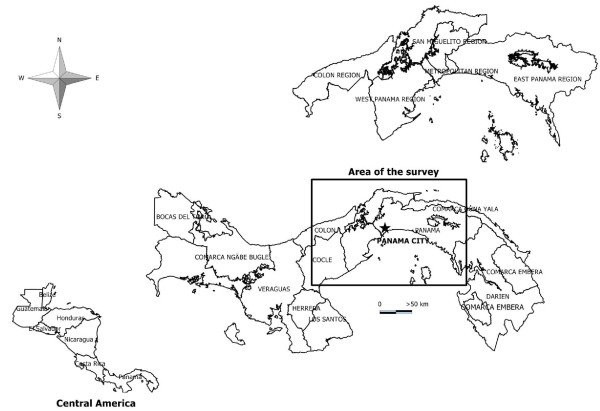
**Republic of Panama.** Political and administrative divisions.

A cross-sectional, descriptive study was conducted in the trans-isthmian zone of the Republic of Panama. The provinces of Panama and Colon, 5 health regions, and the city of Panama (capital of the Republic) are located in this region in which 2,212,722 of the 3,787,511 inhabitants of the country reside, according to estimates produced by the INEC [20]. The country is located at the southernmost end of Central America (Figure 1).

### Universe and sample

The universe included individuals aged 18 years and older who resided in occupied private homes (according to maps produced by the national census for the year 2000). The INEC employed a single-stage, probabilistic, and randomized sampling strategy with a multivariate stratification. The census segments were used as selection strata; samples were calculated separately for urban, rural and indigenous land use areas in the study. The primary sampling units comprised 8–30 private occupied homes, which were first stratified according to the Administrative Political Code of the Republic and then by population size. Internally, they were stratified according to the education level of the study population.

To produce independent estimates for each of the study domains, a sample of independent size was chosen for each domain. We used a level of confidence of 95% and various error levels (5%, 6.5%, 7% or 10%). The sample size was calculated assuming a non-response rate of 10%. A total of 3,505 completed interviews were expected by the end of the survey, which would guarantee the maximum relative error for the average estimates of the variables.

*Inclusion criteria*: Individuals aged 18 and older who permanently resided in private homes in the appropriate census segment; willingness to participate in the survey; and willingness to fast for a period of 8–12 hours.

*Exclusion criteria*: People with severe physical or mental disabilities that prevented them from directly responding to the questions in the questionnaire; lack of fluency in Spanish or a native language; people who resided in private homes that were vacant when the researchers visited the census segment.

### Definitions of variables

Area: Geographic domain where the respondent usually lives (urban, rural or indigenous).

Age: Years from the time of the individual’s birth until the survey was conducted.

Sex: Phenotypical characteristics that distinguish men from women.

Schooling: Grade or school year completed by the interviewee.

Marital status: State of an individual with regard to marriage or free union with a partner (single; married or united; separated or divorced; or widowed).

Sociocultural groups: Cultural, social, economic and ethnic conditions in which an individual lives that influence his interaction with the environment and determine his lifestyle. The following classifications were used: African-American or Afro-Panamanian, Mestizos, Asian, White, Native American or other.

Monthly family income: Total amount of money (in USD) received by the family of the respondent on a monthly basis. USD 600 was used as the cutoff because it is one of the reference points established by INEC (with this amount of money, a Panamanian family can have access to a basic food basket).

People with diabetes: An individual who reported having a medical diagnosis of DM. We also included the individuals who did not have a medical diagnosis but who presented blood glucose values ≥ 126 mg/dl and/or a glycosylated hemoglobin percentage (HbA1c) ≥ 6.5% (≥ 48 mmol/mol) [2,21,22].

People aware of having diabetes: Individuals who reported a medical diagnosis of DM.

People with controlled diabetes: People with diabetes who had values of HbA1c < 7.0% (< 53 mmol/mol) [2,21].

People unaware of having diabetes: Individuals who had fasting blood glucose ≥ 126 mg/dl or HbA1c ≥ 6.5% (≥ 48 mmol/mol) but no reported history of a medical diagnosis.

### Data sources, data collection instruments and procedures

Because this is a population-based survey, the data sources were individuals and biological samples (blood). The data collection instrument was a structured form (survey), developed by researchers from GMI whom are specialists in the department of Chronic Diseases of the MOH and representatives of the Pan American Health Organization in Panama. The planning process for the study included a pilot test to evaluate the methodology, procedures, instruments and the organization of the fieldwork, thus reducing the risk for bias. The instrument was also validated by nationally recognized specialists in the fields of endocrinology, cardiology, nephrology, neurology and public health.

The questionnaire was administered by professionals and students in their final year of Health Sciences education, who were trained by the researchers in interviewing and survey management to standardize the data collection process. In the indigenous areas, the survey administrators were supported by interpreters who spoke the indigenous dialect.

The fieldwork was conducted on weekends. To guarantee an adequate response rate and to ensure that the participants would be fasting, the population segments were visited 15 days before the survey was conducted. Using the spiral scanning technique, we conducted a random survey of people aged 18 years and older residing in occupied housing who agreed to participate in the research (maximum 2 adults per household).

The participants were given a flyer explaining the objectives of the research, confidentiality, the voluntary nature of the study, where to go on the day of the survey, and the fasting requirements (i.e., do not consume food for 8–12 hours or drink alcoholic beverages for 24 hours prior to blood sample collection).

This methodological procedure was repeated one day before (Saturday) the administration of the survey (Sunday) to remind people about the research and to guarantee fasting and participation. On the day of the survey, the researchers confirmed that the participants met the fasting criteria before collecting the blood samples.

To measure serum glucose levels, the blood samples were collected in gel tubes without anticoagulant, and tubes containing Ethylenediaminetetraacetic Acid (EDTA) were used to measure HbA1c. Using high-tech, portable centrifuges, the samples were centrifuged for 10 minutes at 3,500 rpm according to the established protocol. The tubes were placed in containers with frozen packs (2–8°C) to be transferred to the GMI and analyzed at the Central Reference Laboratory in Public Health using the Beckman Synchron CX7 clinical system (multiple wavelength spectrophotometer with diffraction reticle and original reagents). The samples were processed daily, and calibration methods and equipment quality control measures were applied.

### Quality control and data capture

Five trained individuals (1 per health region) administered 3,590 surveys, thereby reducing the risk of error. After the data were collected, 3 of the 5 surveyors performed a quality control check by verifying the data in the 3,590 completed surveys, thus providing a clean database for the analysis.

### Analysis plan

The prevalence estimates from the study sample were calculated as percentages with 95% confidence intervals and a p value. Comparisons were made with the age-adjusted rates for the Panamanian population in 2012 [20]. Logistic regression was used to identify the sociodemographic variables that were significantly associated with DM, and a risk analysis was performed [odds ratio (OR)]. ORs and p values were calculated using 2 × 2 tables, and a value of p ≤ 0.05 was considered statistically significant [23,24].

The data were entered in the CS-Pro 4.0 program in accordance with INEC’s recommendation and processed using SPSS (version 19), Microsoft Excel 2010, Manifold 8 and the free version of Epi info (version 3.5.1).

All of the participants signed an informed consent form. The research was approved by the National Bioethics Committee of the Republic of Panama.

## Results

### General characteristics

The response rate (for both the survey and the fasting blood sampling) was 102.4% (3590/3505), which included 1074 men (29.9%) and 2516 women (70.1%). Of the respondents, 20.5% (735) were young adults (18–29 years), 57.5% (2064) were adults (30–59 years) and 22.0% (791) were seniors (aged 60 and over). The average age of the respondents was 45 years (48 for men, 44 for women) and the median was 44 years (49 for men, 43 for women).

### Prevalence and sociodemographic distribution

Of the participants, 7.3% (262/3590) were aware of having diabetes; 5.7% (204/3590) had fasting glycemic values ≥ 126 mg/dl; 4.3% (156/3590) had HbA1c levels ≥ 6.5% (≥48 mmol/mol); and 2.2% (78/3590) were unaware of having diabetes. Overall, these figures indicated that the estimated prevalence of DM was 9.5% (340/3590). The age-adjusted rate of DM for the 2012 Panamanian population was estimated at 7.7% (Table 1).

**Table 1:** Specific and age adjusted rates of people with diabetes mellitus according to the sociodemographic variables of the research

**Sociodemographic variables**	**Total**		**Specific rate of people with diabetes mellitus**		**Confidence interval (95%)**	**p value**^**a**^	**Age adjusted rate of DM to Panamanian population for 2012**	**People without diabetes mellitus**	
	**Frequency**	**%**	**Frequency**				**%**	**Frequency**	**%**
**Total**	3590	100.0	340	9.5	8.5–10.5	NA	7.7	3250	90.5
**Area**									
Urban	1688	47.0	177	10.5	9.0–12.0	0.0575	7.9^b^	1511	89.5
Rural	1699	47.3	156	9.2	7.8–10.6	0.6148	8.0^b^	1543	90.8
Indigenous	203	5.7	7	3.4	0.9–5.9	0.0038	3.3^b^	196	96.6
**Sex**									
Men	1074	29.9	111	10.3	8.5–12.1	0.2742	8.2	963	89.7
Women	2516	70.1	229	9.1	8.0–10.2		7.9	2287	90.9
**Age** (years)									
18-29	735	20.5	11	1.5	0.0–2.4	0.0000	1.5	724	98.5
30-39	692	19.3	35	5.1	3.5–6.7	0.0000	5.1	657	94.9
40-49	715	19.9	58	8.1	6.1–10.1	0.1884	8.1	657	91.9
50-59	657	18.3	101	15.4	12.6–18.2	0.0000	15.4	556	84.6
60 and over	791	22.0	135	17.1	14.7–19.7	0.0000	17.1	656	82.9
**Sociocultural groups**									
African American (Afro-Panamanian)	757	21.1	90	11.9	9.6–14.2	0.0090	9.5	667	88.1
Mestizo	1937	54.0	168	8.7	7.4–10.0	0.1319	7.0	1769	91.3
Asian	27	0.8	2	7.4	0–16.0	NA	5.8	25	92.6
Native	391	10.9	21	5.4	3.2–7.6	0.0053	5.0	370	94.6
White	431	12.0	51	11.8	8.8–14.8	0.0745	9.3	380	88.2
Others	44	1.2	8	18.2	NA	NA	18.0	36	81.8
Not specified (No response)	3	0.1	0	0.0	NA	NA	NA	3	100.0
**Monthly family income**									
600 USD and more	507	14.1	62	12.2	9.4–15.0	0.0342	9.7	445	87.8
Less than 600 USD	2929	81.6	267	9.1	8.1–10.1		7.6	2662	90.9
Does not know/Does not work	154	4.3	11	7.1	NA	NA	4.1	143	92.9
**Marital status**									
Single	734	20.4	66	9.0	6.9–11.1	0.6543	8.6	668	91.0
Free union or married	2460	68.5	229	9.3	8.2–10.4	0.6229	7.8	2231	90.7
Separated or divorced	225	6.3	19	8.4	4.8–12.0	0.6627	5.0	206	91.6
Widow	163	4.5	26	16.0	10.4–21.6	0.0061	5.9	137	84.0
Not specified (No response)	8	0.2	0	0.0	NA	NA	NA	8	100.0
**Schooling**									
No schooling	186	5.2	10	5.4	2.2–8.6	0.0673	2.3	176	94.6
Elementary	1188	33.1	129	10.9	9.1–12.7	0.0528	7.2	1059	89.1
High school	1499	41.8	125	8.3	6.9–9.7	0.0570	8.0	1374	91.7
University	622	17.3	59	9.5	7.2–11.8	0.9510	9.3	563	90.5
Post-graduate	62	1.7	10	16.1	7.0–25.2	0.1124	15.1	52	83.9
Not specified (No response)	33	0.9	7	21.2	7.3–35.1	NA	NA	26	78.8

^a^P value was carried out among the total number of people with diabetes and without diabetes (according area, sex, age groups, sociocultural groups, monthly family income, marital status and schooling).

^b^Age adjusted rate to Panamanian population for 2010 for urban, rural and indigenous areas.

NA: Not applicable.

A lower prevalence of DM was found in indigenous areas compared with urban and rural areas. With regard to sex, 10.3% of men (111/1074) and 9.1% of women (229/2516) had diabetes (Table 1).

**Figure 2:**
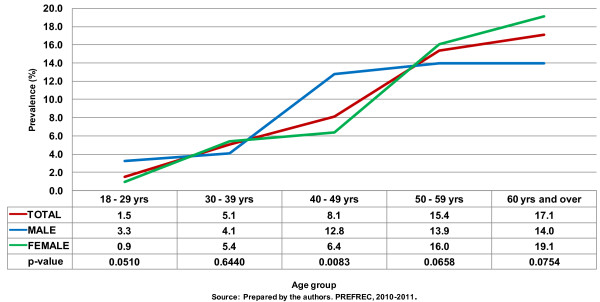
Prevalence of diabetes mellitus (age and sex adjusted rates).

In general, the rate of DM increased in proportion to increasing age. The rate of DM was higher among men than women under 50 years old, but for older individuals, the prevalence of DM was greater among women (Figure 2).

With regard to other sociodemographic variables, the highest prevalence rates were found among Afro-Panamanians [11.9%; (90/757), p < 0.05], widowers [16.0% (26/163; p < 0.05)], those earning 600 USD and above per month [12.2% (62/507; p < 0.05)] and holding post-graduate degrees (16.1%; 10/62), while the lowest prevalence rate was recorded among indigenous groups [5.4%; (21/391), p < 0.05] (Table 1).

The logistic regression revealed relationships between DM and age (p < 0.0001), sex (p < 0.0001), area of residence (p < 0.0001) and sociocultural groups (p = 0.0241). Being Afro-Panamanian and 50 years or older are sociodemographic risk factors associated with DM in Panama, Table 2.

**Table 2:** Sociodemographic risk factors (O.R.) associated with DM in Panama

**Sociodemographic variables**	**O.R.**^**a**^	**Confidence intervals (95%)**
**Total**	NA	
**Area**		
Urban	1.25	0.99–1.57
Rural	0.94	0.74–1.18
Indigenous	0.33	0.14–0.73^b^
**Sex**		
Men	1.15	0.90–1.47
Women		
**Age group** (years)		
18-29	0.12	0.06–0.22^b^
30-39	0.45	0.31–0.66^b^
40-49	0.81	0.60– 1.10
50-59	2.05	1.58–2.65^b^
60 and over	2.60	2.05–3.31^b^
**Socio-cultural groups**		
Afro-Panamanian	1.42	1.09–1.85^b^
Mestizo	0.84	0.66–1.05
Asian	NA	NA
Native	0.52	0.32–0.83^b^
White	1.35	0.97–1.88
Others	NA	
Not specified (No response)	NA	

^a^O.R. was carried out among the total number of people with diabetes and without diabetes in urban, rural and indigenous areas; men and women; age group and sociocultural groups.

^b^p value < 0.0001.

NA: Not applicable.

### Treatment and control

**Figure 3:**
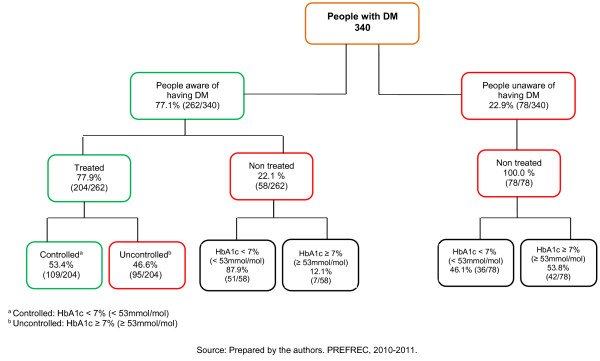
Treatment and HbA1c levels in people aware of having DM and unaware.

Of the 340 people with diabetes, 77.1% (262/340) were aware of having diabetes and 22.9% (78/340) were unaware. In addition, 60.0% (204/340) were receiving treatment and 40.0% (136/340) were not. Only 53.4% (109/204) of the people being treated for DM were controlled, Figure 3.

## Discussion

### General characteristics

This is the first epidemiological study conducted in Panama on the prevalence of cardiovascular risk factors, including DM. A strict methodology was employed to reduce bias and to ensure that the data were statistically representative, high quality, precise and accurate. Among the studies that were consulted, PREFREC was the only study that considered a person’s previous diagnosis of DM (glycemic levels ≥ 126 mg/dl and/or glycosylated hemoglobin ≥ 6.5% (≥ 48 mmol/mol)).

Although the total response rate was 102.4%, the ratio of female–male responses was 2.3:1, with an even higher ratio for those under 40 years of age. This result reflects selection bias, which may be related to the type of sampling strategy used (stratified according to education level); the cultural traditions in the country and the greater acceptance of women participating in population-based research; or the requirement to abstain from alcohol for 24 hours before the survey, which may have lowered the participation rate among men.

Using age-adjusted rates allowed us to control for confusion bias. For all of the sociodemographic variables, the age-adjusted rates were lower than the unadjusted rates. The effect was most notable for marital status and schooling. There were no differences between the unadjusted rates and the age- or sex- adjusted rates.

### Prevalence and sociodemographic aspects

The prevalence of DM found by PREFREC (9.5%; CI 8.5 - 10.5) is comparable with other studies [3-5,8,25-29] and allows us to estimate that in Panama in 2012, there were 238,367 persons with DM. Of these, 144,690 resided in the trans-isthmian area of the country, where 60.4% of Panamanians 18 years and older reside [20].

The percentage of people aware of having diabetes was higher than the value estimated in 2007 by the National Survey on Health and Quality of Life (5.8%) in the same area [30]. The high prevalence estimated by PREFREC justifies allocating government resources to comprehensive care for persons with DM because of the high cost of treating this disease and the benefits of preventing complications.

In Panama, 1 in every 5 persons with DM is unaware of their condition. This level of unawareness is comparable to other countries in the region [15,29] but lower than the global estimate of 50% reported by the International Diabetes Federation [5].

The logistic regression analysis identified the sociodemographic variables that were significantly associated with DM in the Panamanian population.

There was an epidemiological trend toward higher rates of DM with increasing age. Although the bivariate analysis (OR) determined that being younger than 40 years old is a protective factor against DM, the presence of DM in individuals between 18 and 29 years old suggests that risk factors (such as obesity, physical inactivity and unhealthy diets rich in fats and sugars) are present from the earliest stages of life. Thus, we must consider multiple causes in the epidemiological profile of DM.

On the other hand, the OR indicates that age is a risk factor for DM for individuals over age 50. Considering that Panama’s population pyramid tends to be inverted and the life expectancy for Panamanians has increased to 78 years [20], the increase in the number of individuals with DM is expected to continue in the coming years, as well as the number of deaths caused by this disease.

According to the analysis of risk (OR), being native and living in the indigenous area are protective factors against DM. This result suggests that there are certain social, cultural and environmental determinants of DM related to indigenous lifestyles (e.g., less sedentary lifestyle and less Westernized eating habits) and justifies the need for conducting other investigations in this population.

The prevalence of DM among Afro-Panamanians is high, and furthermore, the OR indicates that being Afro-Panamanian is a risk factor for DM in Panama. In addition to the genetic component, the influence of dietary habits in this group could be a decisive factor in this observed high prevalence: high consumption of fatty meals is deeply rooted in the Afro-Caribbean culture and contributes to the development of the disease [31].

Although the result was not statistically significant, there were more men with DM than women [29]. However, among those older than 60, the prevalence was higher among women, which coincides with the projections made by other authors, who suggest that this sex/age pattern may occur because senior populations have a greater number of women than men [19]. On the other hand, the higher prevalence of DM among older women may increase the probability of death among women compared with men, which would explain the increase in the female to male ratio in DM mortality that has been observed in the country over the last 5 years (1.3:1) [18].

The fact that there is a higher prevalence in the subgroup of widows could be related to the relatively older age of widowed persons in addition to the possible effect of loneliness as a source of emotional tension and a cause of neglect in self-care.

The prevalence of DM was directly proportional to monthly family income. The increase in prevalence among individuals with higher incomes could be explained by their greater purchasing power, which could increase caloric intake and reduce physical activity. Individuals in this group may own automobiles for transportation and may have more educational opportunities that enable them to find jobs requiring less physical activity.

The differences in education seem to follow the trend in income. Individuals with higher education have higher incomes, which would explain the higher prevalence. Therefore, although it might seem that a higher educational level would be a protective factor because an educated person would have greater knowledge about the impact of lifestyle factors on health and more accurate risk perception [32], the consequences of a higher income (mentioned previously) exceed the benefits afforded by a higher educational level.

### Treatment and control

Interestingly, 53.4% (109/204) of those who were aware of having diabetes had an HbA1c value < 7.0% (< 53 mmol/mol), which indicates appropriate metabolic control [21,26,33,34]. This figure compares favorably with the results from other nations [28,35]. However, 46.6% (95/204) of DM patients have not stabilized the disease, and undoubtedly, we must strive to reduce this figure.

It is worth noting that among the individuals who were aware of having diabetes, only 77.9% were receiving treatment (medication). Although the cause was not investigated, we can report that 2 out of every 10 persons with a medical diagnosis of DM do not comply with the current international guidelines for receiving treatment from the time of diagnosis [21,33].

Additionally, 22.1% (58/262) of the people who were aware of having diabetes were not taking medication for their disease. Of these, 87.9% (51/58) had an HbA1c value < 7.0%, and 46.1% (36/78) of the people unaware of having diabetes also had values in this range. These results suggest that although these people had altered glucose metabolism at the moment of the survey, there had not been sufficient time for sustained hyperglycemia to generate glycosylation greater than 7%. Another possibility is that these individuals had less aggressive diabetes (given the slight elevation in their glycemic levels), so they had not experienced symptoms that prompted them to seek medical attention and receive a diagnosis. In addition, other risk and protective factors related to lifestyle (type of food, physical activity, etc.) and demographics should also be considered as explanatory variables.

These statistics should be evaluated by health professionals because if these individuals are not diagnosed and do not receive timely medical treatment, their HbA1c values will eventually exceed 7%, producing macro- and micro-vascular complications. Over the middle and long term, these health problems will increase the economic and social burden of the disease in the country.

## Conclusions

This study found that the prevalence of DM was 9.5%. Because this research included a representative sample of urban, rural and indigenous populations, and because there have been no previous studies on this topic, these results can be used as a country public health indicator.

Being Afro-Panamanian and 50 years of age or older are sociodemographic risk factors for DM in Panama, whereas being native, 39 years of age or younger and living in an indigenous area are protective factors.

The Panamanian health system must make an effort to reach the 22.9% of people who are unaware that they have DM and are thus not receiving treatment. This effort would reduce health complications and improve the quality of life for individuals with the disease, so we recommend actively searching for such cases.

This information is essential for developing integrated care programs targeting people with DM.

## Abbreviations

DM: Diabetes Mellitus; INEC for its acronym in Spanish: National Institute of Statistics and Census; PREFREC for its acronym in Spanish: Survey on Risk Factors Associated to Cardiovascular Disease; GMI: Gorgas Memorial Institute for Health Research; MOH: Panamanian Ministry of Health; EDTA: Ethylenediaminetetraacetic Acid; HbA1c: Glycosylated hemoglobin; USD: United States Dollars; O.R.: Odds Ratio.

## Competing interests

The authors declare that they have no competing interests.

## Authors’ contributions

AMcD researched, analysis and interpretation of data, wrote the manuscript and gave final approval of the version to be published. JM wrote the manuscript and gave final approval of the version to be published. CC analysis and interpretation of data. AR have been involved in drafting the manuscript and revising it critically for important intellectual content. AC researched data and has been involved in drafting the manuscript. All authors read and approved the final manuscript.
